# MXD1 localizes in the nucleolus, binds UBF and impairs rRNA synthesis

**DOI:** 10.18632/oncotarget.11766

**Published:** 2016-08-31

**Authors:** Maria del Carmen Lafita-Navarro, Rosa Blanco, Jorge Mata-Garrido, Judit Liaño-Pons, Olga Tapia, Lucía García-Gutiérrez, Eva García-Alegría, María T. Berciano, Miguel Lafarga, Javier León

**Affiliations:** ^1^ Instituto de Biomedicina y Biotecnología de Cantabria (IBBTEC), CSIC-Universidad de Cantabria, and Department of Molecular Biology, University of Cantabria, Santander, Spain; ^2^ Department of Anatomy and Cell Biology and Centro de Investigación en Red sobre Enfermedades Neurodegenerativas (CIBERNED), University of Cantabria-IDIVAL, Santander, Spain; ^3^ Present address: Department of Cell Biology, UT Southwestern Medical Center, Dallas, Texas, USA; ^4^ Present address: Stem Cell Hematopoiesis Group, Cancer Research UK Manchester Institute, University of Manchester, Manchester, United Kingdom

**Keywords:** MXD1, UBF, nucleolus, pre-rRNA, transcription regulation

## Abstract

MXD1 is a protein that interacts with MAX, to form a repressive transcription factor. MXD1-MAX binds E-boxes. MXD1-MAX antagonizes the transcriptional activity of the MYC oncoprotein in most models. It has been reported that MYC overexpression leads to augmented RNA synthesis and ribosome biogenesis, which is a relevant activity in MYC-mediated tumorigenesis. Here we describe that MXD1, but not MYC or MNT, localizes to the nucleolus in a wide array of cell lines derived from different tissues (carcinoma, leukemia) as well as in embryonic stem cells. MXD1 also localizes in the nucleolus of primary tissue cells as neurons and Sertoli cells. The nucleolar localization of MXD1 was confirmed by co-localization with UBF. Co-immunoprecipitation experiments showed that MXD1 interacted with UBF and proximity ligase assays revealed that this interaction takes place in the nucleolus. Furthermore, chromatin immunoprecipitation assays showed that MXD1 was bound in the transcribed rDNA chromatin, where it co-localizes with UBF, but also in the ribosomal intergenic regions. The MXD1 involvement in rRNA synthesis was also suggested by the nucleolar segregation upon rRNA synthesis inhibition by actinomycin D. Silencing of MXD1 with siRNAs resulted in increased synthesis of pre-rRNA while enforced MXD1 expression reduces it. The results suggest a new role for MXD1, which is the control of ribosome biogenesis. This new MXD1 function would be important to curb MYC activity in tumor cells.

## INTRODUCTION

MXD1 (also known as MAD1) is a transcription factor belonging to the MXD family of proteins [1]. The MXD family (composed of MXD1, MXI1/MXD2, MXD3, MXD4 and MNT) is part of the MYC-MAX-MXD network of transcription factors. These factors contain the b-HLH-LZ domain by which they bind to E-boxes in the promoter of their target genes to regulate their transcription [2–4].Whereas MYC-MAX activates transcription, MXD1-MAX represses transcription forming complexes with histone deacetylases. Therefore, MXD1 acts as antagonist of MYC transcriptional activity [5–7]. Conversely to MYC, MXD1 is readily expressed in resting and differentiated cells [6, 8, 9]. Moreover, MXD1 inhibits cell proliferation and antagonize MYC transforming activity [10–12].

The nucleolus is the nuclear factory for rRNA synthesis, posttranscriptional processing of pre-rRNA and assembly of preribosomal particles. It is organized around clusters of tandem repeats of RNA genes (rDNA) that encode the rRNAs 28S, 18S and 5.8S. The nucleolar structure, composed of fibrillar centers (FCs), dense fibrillar component (DFC) and granular component (GC), reflects the sequential steps of ribosome biogenesis [13–15]. The FC concentrates components of the transcription machinery, such as RNA polymerase I and UBF (Upstream Binding Factor), an essential transcription factor of ribosomal genes [16]. The DFC partially surrounds FCs and contains transcriptionally active ribosomal genes and numerous RNA-binding proteins. RNA pol I transcription takes place in the FCs and in the DFC, with further posttranscriptional processing of pre-rRNA occurring within the DFC to give rise to the mature rRNAs [17, 18]. The GC is where the 40S and 60S ribosome subunits are assembled [19, 20].

MXD1 was described as nuclear in fibroblasts [1] and quiescent keratinocytes [21], but the subcellular localization of endogenous MXD1 is unknown for most cell types. Here we report that MXD1 localizes in the cell nucleolus and describe an interaction of MXD1 with UBF. In addition, we show that MXD1 binds to rDNA and regulates rDNA transcription.

## RESULTS

### MXD1 localization in the nucleolus

**Figure 1:**
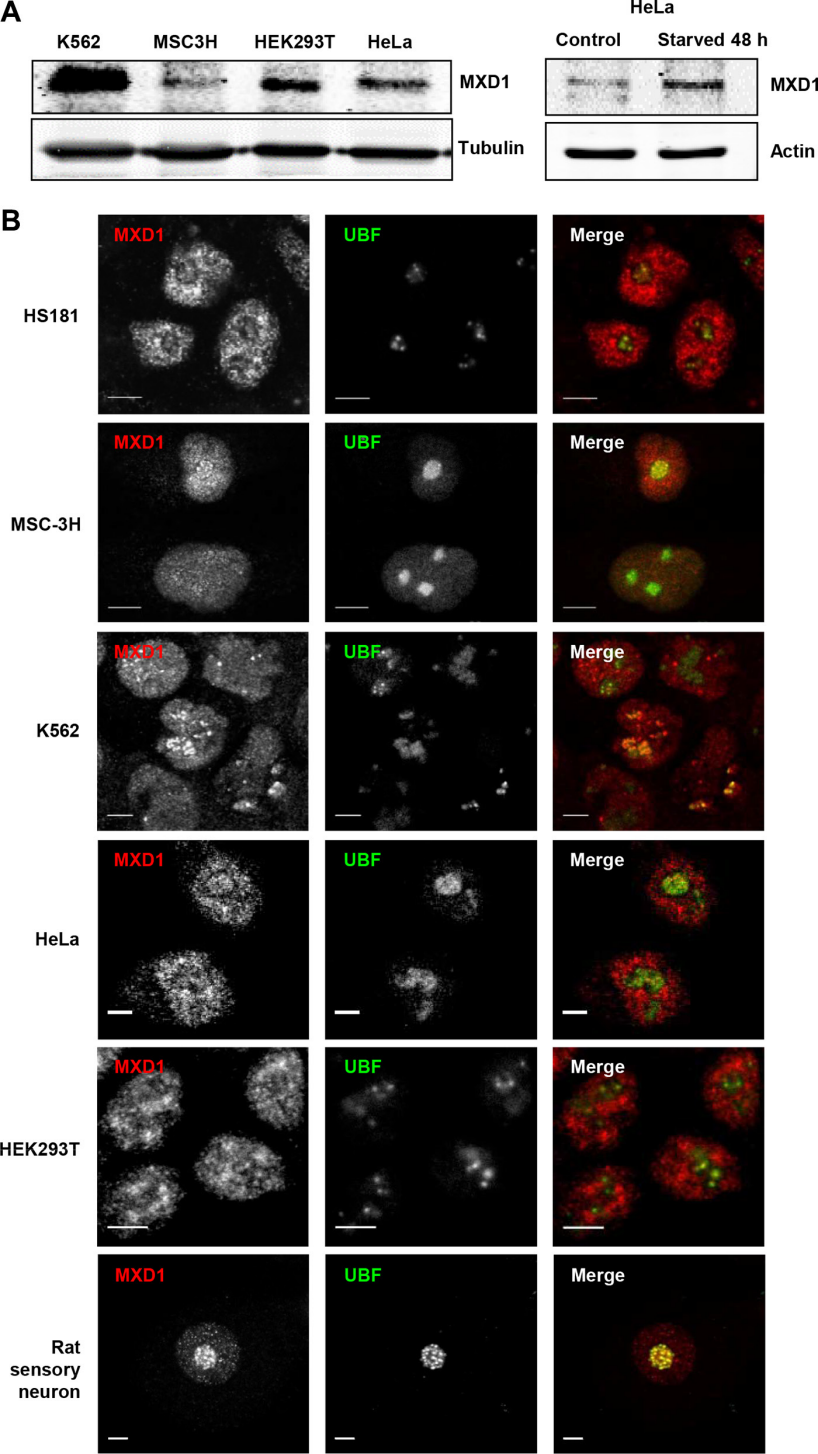
Endogenous MXD1 localizes in the nucleolus (**A**) MXD1 protein expression in growing cells from different cell lines used in this work. MXD1 levels were analyzed by immunoblot in the indicated cell lines (left panel). The right panel shows MXD1 expression in HeLa cells deprived of serum for 48 h. The expression of β-tubulin or α-actin levels were determined as a control for protein loading. (**B**) MXD1 localization in the nucleolus. Cells from the indicated human cell lines and rat sensory neurons were double immunolabelled for MXD1 (red) and UBF (green). MXD1 showed a nuclear distribution, including the nucleolus where colocalized with UBF. Scale bars: 5 μm.

**Figure 2:**
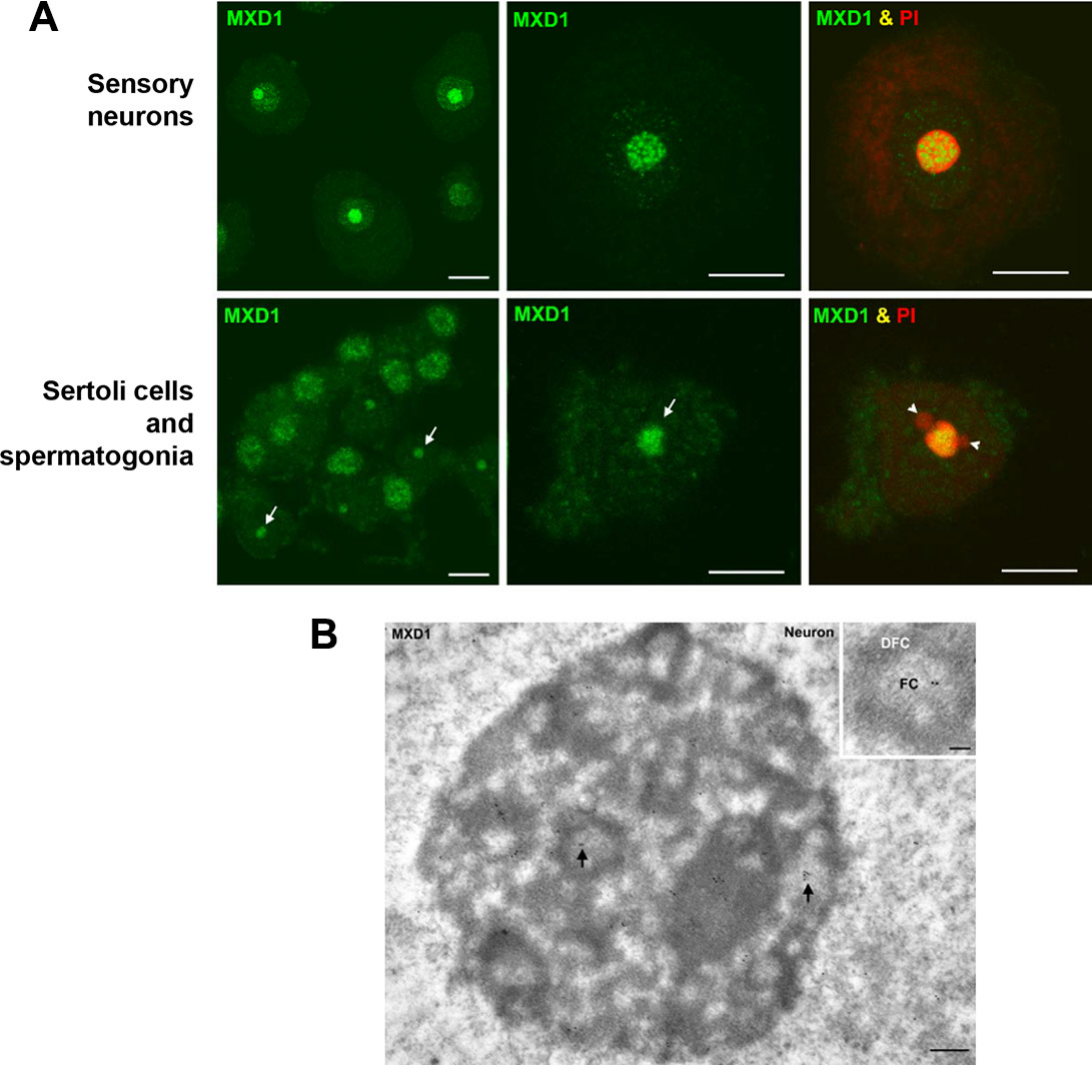
MXD1 expression in rat primary cells (**A**) Immunofluorescence for MXD1 in sensory neurons and seminiferous tubes. In dissociated neurons from rat sensory ganglion MXD1 appeared concentrated in the nucleolus and in nucleoplasmic microfoci. Within the nucleolus, MXD1 aggregated in numerous large foci with the typical pattern of FCs. The prominent neuronal nucleolus is clearly identified by the accumulation of rRNAs stained with PI. Dissociated cells from seminiferous tubes illustrated numerous spermatogonia, with an intense nuclear signal of MXD1, and several Sertoli cells with a nucleolar pattern of MXD1 immunoreactivity (arrows). Co-staining with PI revealed the typical tripartite structure formed by the Sertoli cell nucleolus and two associated masses of perinucleolar heterochromatin (arrowheads). Scale bars: 10 μm. (**B**) Immunogold electron microscopy of MXD1 in a neuronal nucleolus. Tissue fragments from sensory ganglion were immunolabeled with the anti-MXD1 antibody and then with a secondary antibody conjugated with 10 nm gold particles. Immunogold particles decorated fibrillar centers (arrows) and some particles were also observed in the associated dense fibrillar component. Scale bar: 250 nm. Inset. Higher magnification of a fibrillar center (FC) surrounded by the dense fibrillar component (DFC). Note the immunolabeling of the FC. Scale bar: 150 nm. No immunogold particles were detected in control samples without primary antibody.

**Figure 3:**
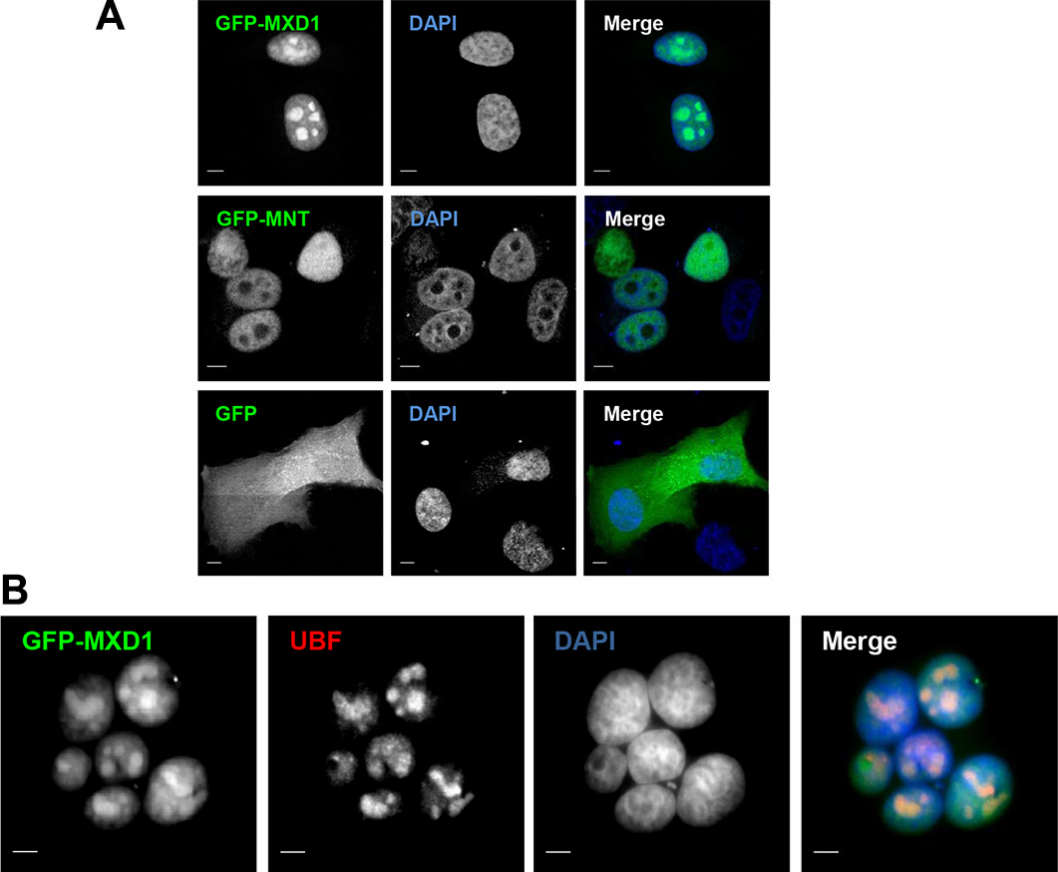
Ectopic MXD1 localizes in nucleoli (**A**) Nucleolar localization of transfected MXD1. HeLa cells transfected with the GFP, GFP-MNT and GFP-MXD1 constructs (green). Whereas GFP-MXD1 protein localized in the nucleolus, GFP-MNT showed a nuclear staining but was absent from the nucleolus. (**B**) HeLa cells were transfected with the GFP-MXD1 construct and 24 h after transfection immunofluorescence was performed for endogenous UBF as described in Figure 1 with a secondary antibody conjugated with Texas Red. Nuclei were stained with DAPI (blue). The images were acquired by confocal (A) or epifluorescence (B) microscopy. Scale bars: 5 μm.

We performed immunofluorescence studies in proliferating embryonic stem cells HS181, mesenchymal stem cells MSC-3H, myeloid leukemia K562 cells, embryonic kidney HEK293T cells, and cervical cancer HeLa cells. Although it is reported that MXD1 is mostly expressed in quiescent cells or differentiated cells, we found low but detectable MXD1 protein levels in proliferating cells of the above cell lines (Figure 1A), although serum deprivation resulted in increased MXD1 expression, as described [22] (Figure 1A). We chose some of these cell lines with higher MXD1 expression to study its subcellular localization. As shown in Figure 1B, MXD1 was localized in the nucleus in all cell lines but, surprisingly, it was also concentrated in the nucleolus in a relevant proportion of cells (70% to 100% depending on the cell line). The nucleolar distribution of MXD1 was confirmed by its co-localization with UBF (Figure 1B), a nucleolar marker of FCs [13, 23]. The nucleolar localization for MXD1 is so far unreported and we set out to confirm this. Analysis of the fluorescence intensity profiles of UBF and MXD1 in cell lines and neurons demonstrated this co-localization (Supplementary Figure S1). We also asked if the subnuclear localization of MXD1 observed in cells in culture also occurred in primary tissue cells. We analysed by immunofluorescence Sertoli and germinal cells from rat seminiferous tube and rat sensory ganglion neurons. MXD1 was concentrated in the nucleolus, with a weaker nucleoplasmic signal, in post-mitotic Sertoli cells and neurons (Figure 2A). The nucleolar localization of MXD1 was confirmed in preparations co-stained with propidium iodide (Figure 2A), and its preferential concentration in FCs of neurons was demonstrated by co-localization with UBF (Figure 1A, lower panel). Moreover, immunogold electron microscopy confirmed the localization of MXD1 in FCs and associated DFC of the neuronal nucleolus (Figure 2B). To further confirm this subnuclear distribution, we transfected HeLa cells with a GFP-MXD1 construct. As controls, we also transfected a GFP-MNT and GFP constructs. Following 24 h of transfection, the transfected GFP-MXD1 protein was diffusely distributed in the nucleoplasm, but concentrated in the nucleolus (Figure 3A). The nucleolar localization of exogenous GFP-MXD1 was confirmed by immunofluorescence with the anti-UBF antibody, showing the co-localization of GFP and UBF signals (Figure 3B).

**Figure 4:**
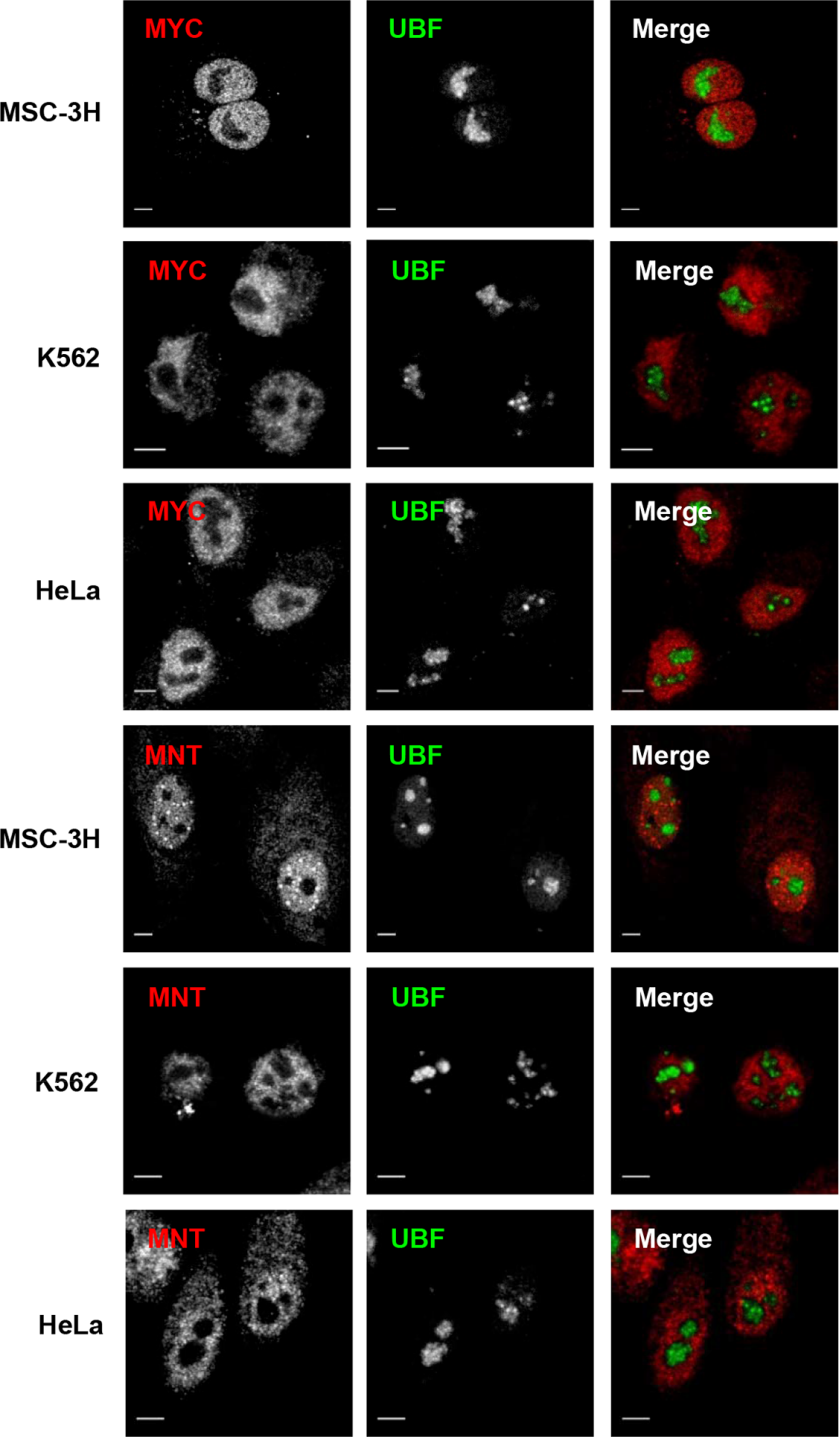
Nucleoplasmic but not nucleolar localization of MYC and MNT (**A**) Cells from the cell lines indicate at the left were co-immunostained for UBF and MYC or MNT. Confocal merged images show the lack of co-localization of MYC and MNT with the nucleolus in all cell lines. Scale bars: 5 μm.

We next studied whether the nucleolar distribution of MXD1 was extensible to MYC and another member of the MXD family as MNT. In sharp contrast to MXD1, both MYC and MNT were present in the nucleoplasm but excluded from the UBF-immunolabeled nucleoli in most cells (Figure 4). Fluorescence intensity line profiles confirmed the absence of MYC and MNT in the nucleolus (Supplementary Figure S2). Moreover, overexpressed MNT did not accumulate in the nucleolus, as shown in HeLa cells transfected with a GFP-MNT construct (Figure 3A).

**Figure 5:**
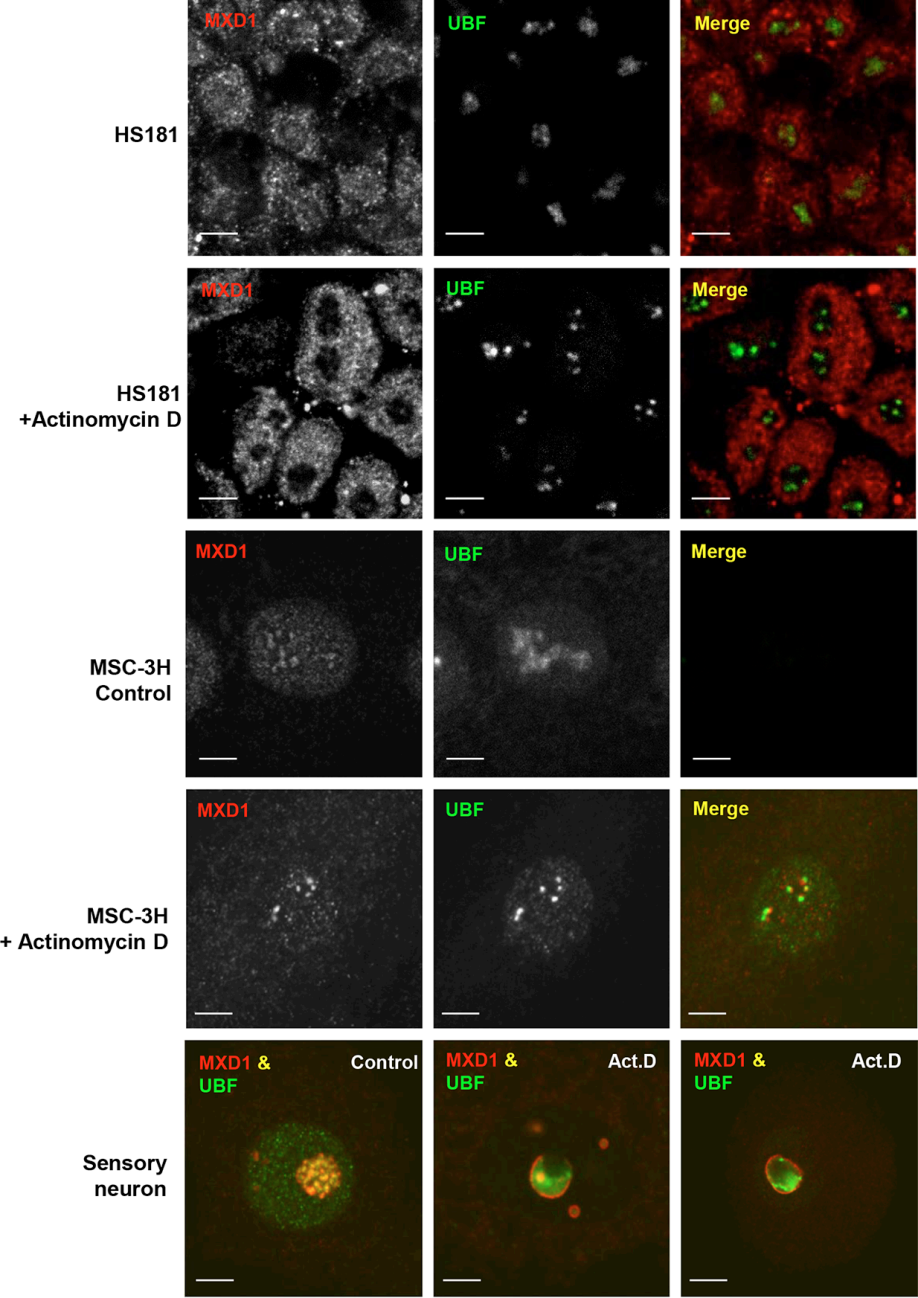
Nucleolar segregation of MXD1 by actinomycin D treatment Control and actinomycin D-treated cells (50 ng/ml for 1 h) from HS181 and MSC-3H cell lines were co-immunostained for MXD1 and UBF. In control HS181 and MSC-3H cells, MXD1 colocalizes with UBF in nucleoli. Inhibition of nucleolar transcription with actinomycin D induces depletion of MXD1 in HS181 nucleoli. In actinomycin D-treated MSC-3H cells, both MXD1 and UBF appear segregated at the nucleolar periphery as juxtaposed perinucleolar caps. In a control rat sensory ganglion neuron, UBF and MXD1 colocalize in numerous fibrillar centers. In contrast, fibrillar centers tend to disappear and MXD1 and UBF segregate upon systemic actinomycin D (Act.D) treatment (300 μg/k for 3 h). Thus, whereas MXD1 segregates as a thin ring around of both the nucleolus and smaller nucleolar fragments and colocalizes with UBF in residual fibrillar centers, the bulk of UBF tends to segregate as a large mass at the nucleolar periphery. Scale bars: 5 μm.

The co-localization of MXD1 and UBF in the FCs opened the possibility that MXD1 might be regulating rDNA transcription. To test this, we treated HS181 and MSC-3H cells with actinomycin D at low concentration (50 ng/ml for 1 h), and performed co-immunostaining for MXD1 and UBF. Under these conditions, actinomycin D inhibits RNA pol I but not RNA pol II and RNA pol III [24, 25]. We also treated rats systemically with actinomycin (300 μg/kg for 3 h) and analysed the nucleolar reorganization in sensory neurons from trigeminal ganglia. Interestingly, we found that RNA pol I inhibition interfered with the nucleolar targeting of MXD1 in all cases, as a MXD1-free nucleolar area appeared after treatment with actinomycin D, meanwhile the nucleoplasmic signal of MXD1 remained unchanged (Figure 5). In MSC-3H cells and sensory neurons, MXD1 was segregated at the nucleolar periphery, forming typical perinucleolar caps or rings in confocal images (Figure 5) [26]. This effect was associated with disruption of FCs and peripheral segregation of the UBF, suggesting that MXD1 localization in FCs correlates with ongoing rRNA synthesis and raising a possible role of this transcription factor in the regulation of rDNA transcription.

### Interaction between MXD1 and UBF

**Figure 6:**
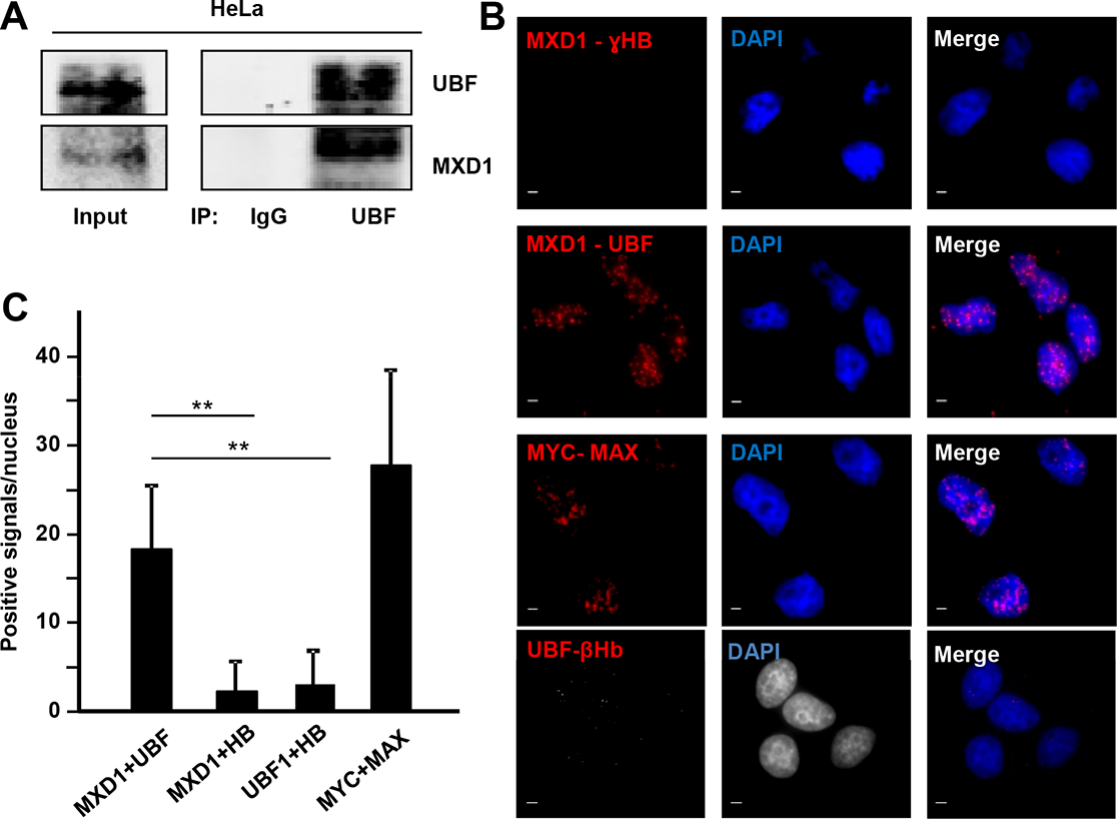
MXD1 and UBF interaction (**A**) Co-immunoprecipitation of MXD1 and UBF in lysates of HeLa cells. Cells were serum-deprived for 48 h and immunoprecipitation of UBF was performed, followed by immunoblot against MXD1 and UBF. (**B**) *In situ* PLA in growing HeLa cells to test MXD1-UBF interaction. The pairs of antibodies used were anti-MXD1 and anti-UBF, anti-MYC and anti-MAX (positive control), anti-MXD1 and anti-ɣ-Hemoglobin (ɣHB) (negative control) and anti-UBF and anti-β-hemoglobin (negative control). Red dots showed the MXD1-UBF interaction. DAPI staining of DNA was used to detect cell nuclei. Scale bars: 5 μm. (**C**) Quantification of PLA signals. PLA positive signals per nuclei were quantified using ImageJ software. At least 200 nuclei were counted for each experimental condition. Data are mean ± s.e.m **P < 0.01.

As UBF and MXD1 co-localized in the FCs of nucleolus, we asked whether MXD1 and UBF interacted in the nucleolus. We first performed immunoprecipitation assays in HeLa cells. The cells were deprived from serum for 48 h to augment MXD1 expression and the lysates were immunoprecipitated with anti-UBF antibodies. Immunoblot analysis showed that MXD1 was present in the immunoprecipitates, indicating a UBF-MXD1 interaction (Figure 6A). Similar results were obtained in cells transfected with UBF-Flag expression vector (not shown). Next, we asked whether this interaction takes place in the nucleoli of the cells. To analyse this, we performed an *in situ* proximity ligation assay (PLA) in HeLa cells. As shown in Figure 6B *in situ* PLA signal was positive with antibodies against MXD1 and UBF. This interaction was higher in discrete areas of the nuclei, likely corresponding to the nucleoli. No interaction was detected in the cytoplasm, serving as a negative control. Interaction was also observed between MYC and MAX (positive control), but no signal was detected when we performed the assay with antibodies against MXD1 or UBF and hemoglobins (negative controls). Signal quantification indicated that MXD1 and UBF interact but less than MYC-MAX (Figure 6C). Taken together, these results suggest that MXD1 and UBF are interacting at the site of the rRNA synthesis in the nucleolus.

**Figure 7:**
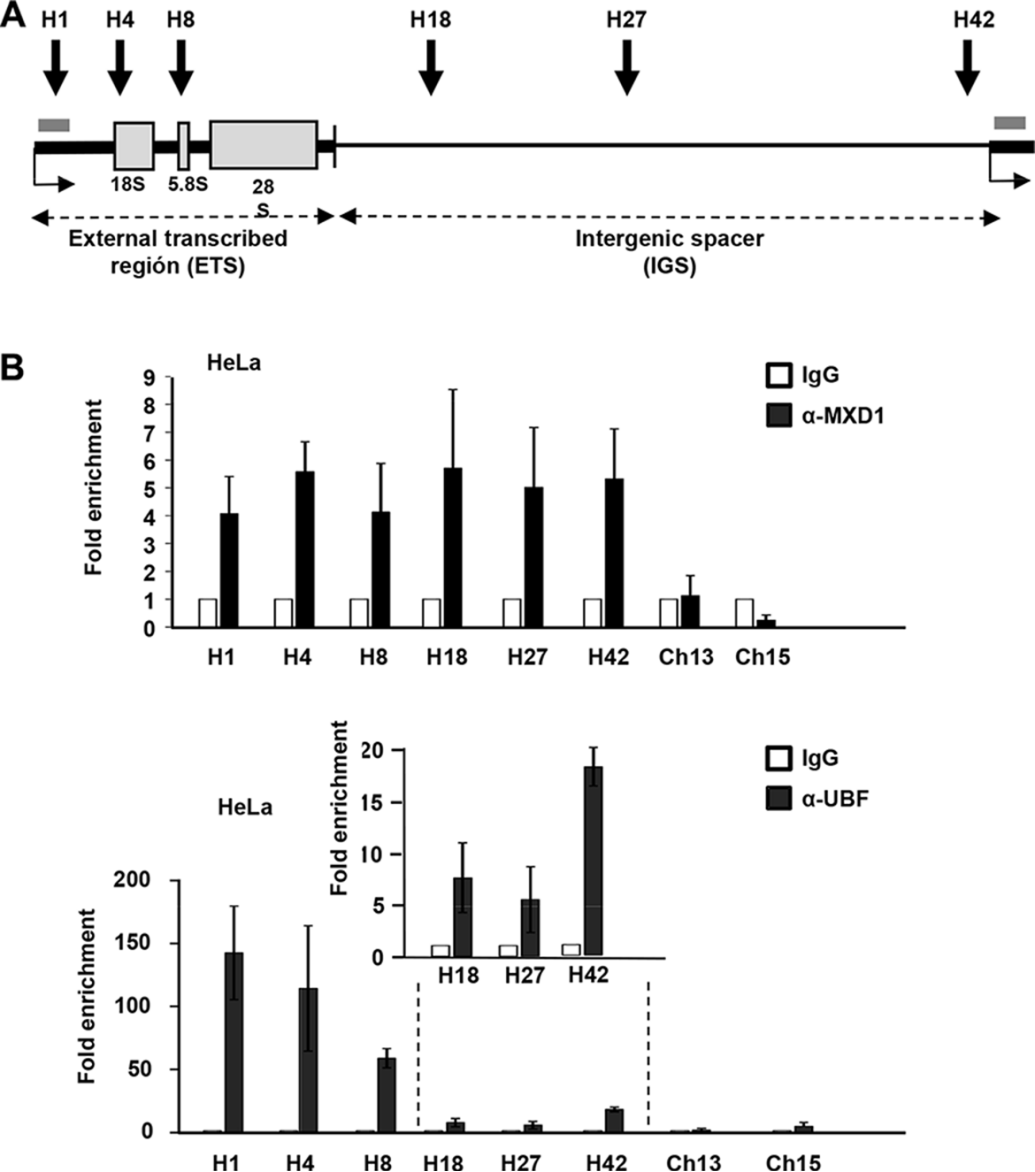
MXD1 binding to rDNA chromatin (**A**) Schematic representation of a rDNA repeat showing the sequences of the three mature rRNAs (grey boxes), the introns (thick line) and the intergenic region (IGS, thin line). The grey bar represents the amplicon used for pre-rRNA determination by RT-qPCR. (**B**) ChIP of MXD1 and UBF in HeLa cells deprived of serum for 48 h. The amplicons H1-H42 cover different regions of the rDNA gene and intergenic regions [27]. Data are mean values from four ChIP experiments. The inset shows the signals of UBF in the H18-H42 amplicons.

As MXD1 localized in the FCs of nucleoli, we hypothesized that it might be taking part in the regulation of rRNA synthesis. We first asked whether MXD1 was bound to the rRNA genes. The human rRNA genes are organized in clusters of ~43 kb repeats in tandem distributed among five different chromosomes (chromosome number 13, 14, 15, 21 and 22). We performed a chromatin immunoprecipitation assay (ChIP) of MXD1 on the rDNA in HeLa cells. We studied MXD1 binding to regions already analysed for MYC binding [27] in the transcribed region and in the intergenic spacer (Figure 7A). We performed this analysis in the chromatin of HeLa cells after 48 h of serum deprivation, in order to increase the levels of MXD1. As negative controls, we tested two amplicons mapping in the long arm of chromosomes 13 and 15 (i.e., the opposite arm to where rDNA genes map). The results showed that MXD1 was bound throughout the entire rDNA repeat, in the same regions already reported as bound to MYC [27, 28] (Figure 7B). As a positive control, we performed ChIP analysis for UBF, which bound to the rDNA transcribed regions (H1, H4, H8) and less in the IGS (H18, H27, H42) [27, 29] (Figure 7B). As expected, UBF binding was much stronger than that of MXD1. Similar results were found in HEK293T cells (Supplementary Figure S3).

**Figure 8:**
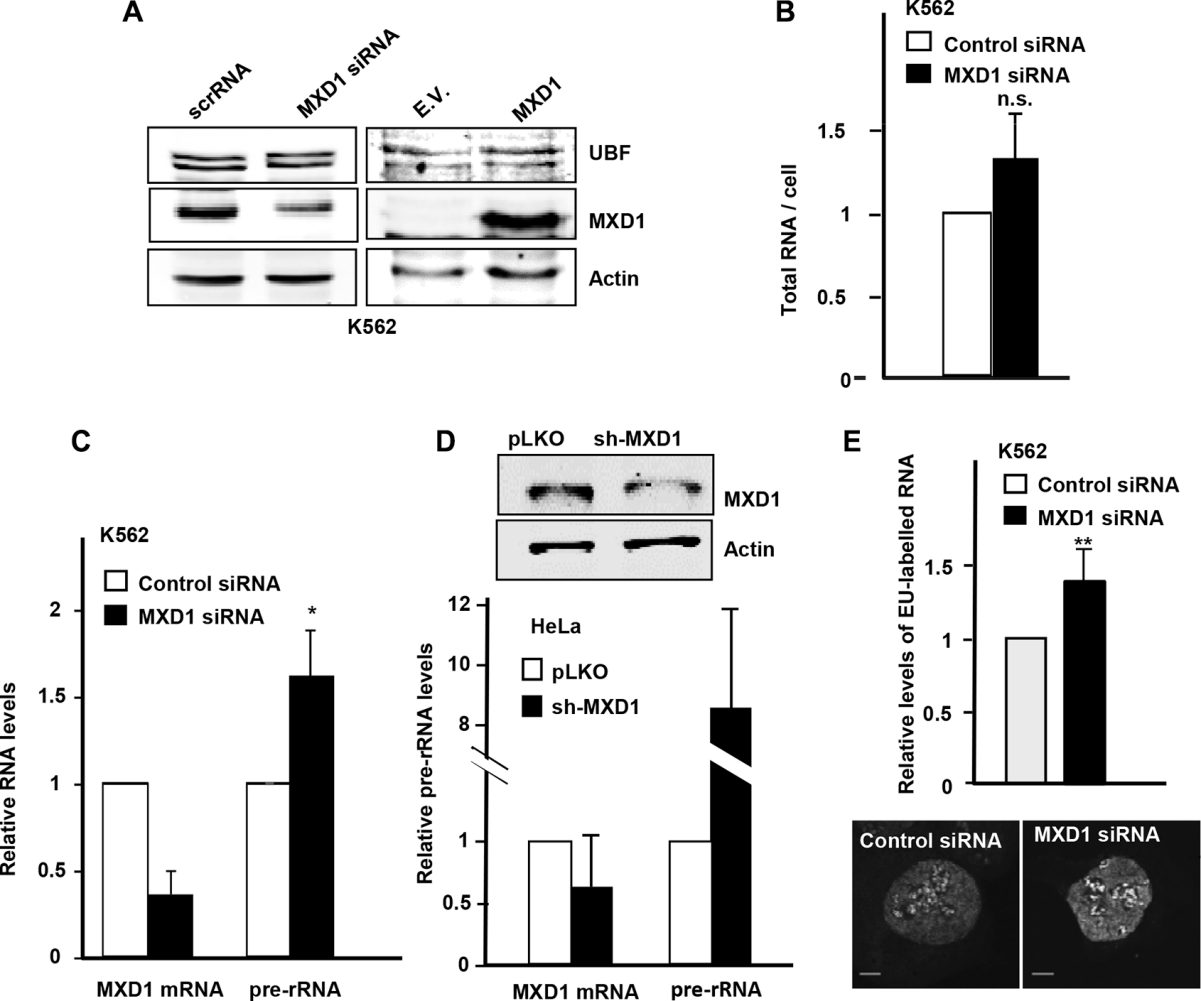
Effect of MXD1 depletion on rRNA levels (**A**) Left panel: K562 were transfected with MXD1 siRNA or control siRNA. 72 h after transfection, MXD1 and UBF levels were analysed by immunoblot. Right panel: K562 were infected with GFP-MXD1 lentivirus or empty vector lentivirus (E.V.). 5 days after infection MXD1 and UBF levels were analysed by immunoblot. The levels of α-actin were determined as a control of protein loading. (**B**) Total RNA per cell in K562 cells 72 h after MXD1-siRNA transfection. (**C**) Expression of 45S pre-rRNA upon MXD1 knock-down in K562 cells. Cells were transfected with MXD1 siRNA and 72 h after transfection the RNA was analyzed by RT-qPCR. Data are mean ± s.e.m. from four independent transfections. (**D**) Expression of 45S pre-rRNA upon MXD1 knock-down in HeLa cells. Cells were transfected with a MXD1 short-hairpin vector and after 15 days of selection with puromycin, RNA was prepared and the expression of 45S pre-rRNA was analyzed by RT-qPCR. Data are mean values from two independent transfections ± s.e.m. The depletion of MXD1 protein was confirmed by immunoblot (upper panel). (**E**) Nascent EU-labelled rRNA after a 15 min EU pulse in K562 cells transfected with MXD1 siRNA. Signals from 100 cells were quantified using ImageJ software. Two representative cells are shown in the bottom panel. Scale bars: 5 μm. Error bars are s.d. **P < 0.001.

**Figure 9:**
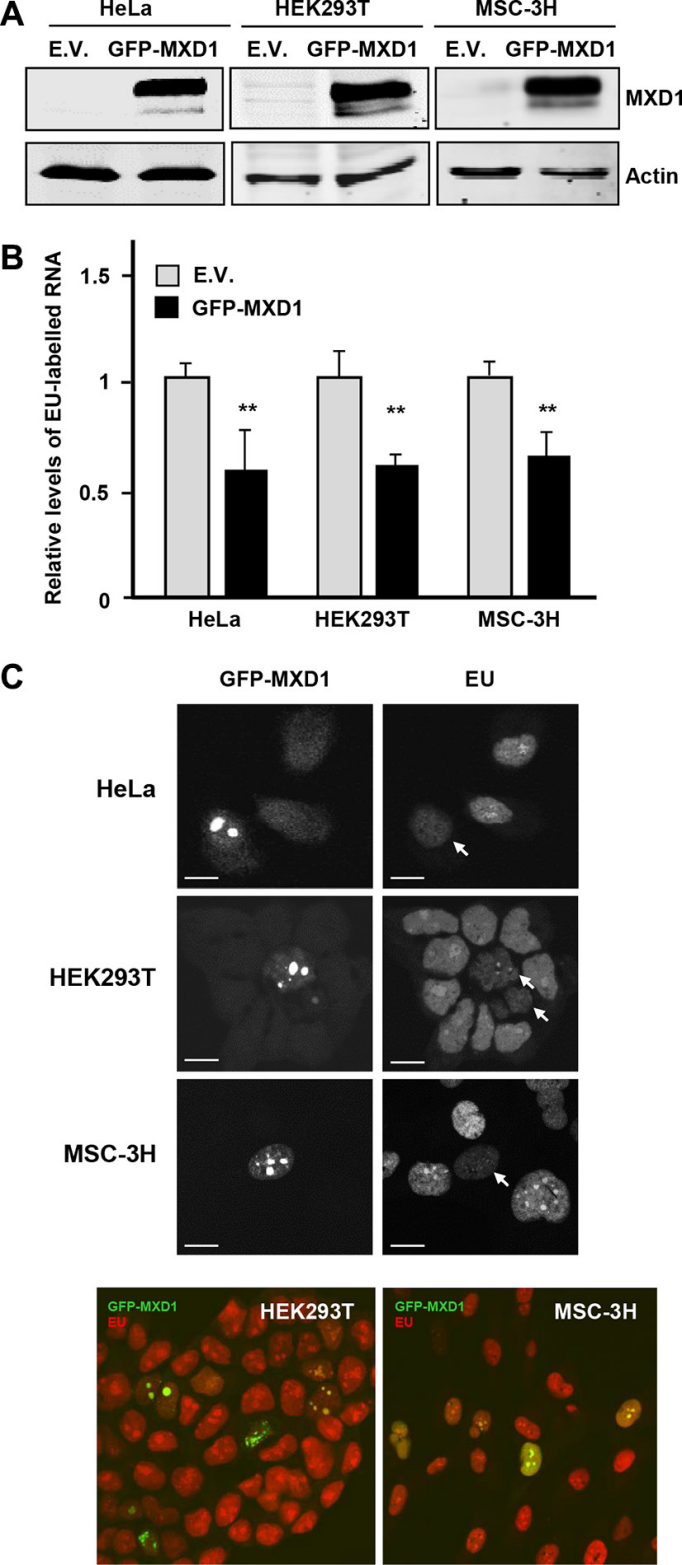
Effect of MXD1-enforced expression on rRNA synthesis (**A**) Overexpression of GFP-MXD1 protein after transfection of GFP-MXD1 in HeLa and HEK293T, and lentiviral transduction of MSC-3H cells. The GFP-MXD1 protein was detected by immunoblot 48 h after transfection or virus infection. α-actin levels were determined as a control for protein loading. (**B**) Nascent EU-labelled rRNA after a EU pulse of 45 min in the indicated cell lines transfected with an expression vector for GFP-MXD1 or the empty vector (E.V., pReceiverLv103 vector). Error bars are s.d. **P < 0.001. (**C**) Representative images showing a transfected (GFP-positive) cell and several untransfected cells. Note the accumulation of GFP-MXD1 in the nucleoli and the reduced EU labelling (arrows). Representative confocal merged images of HEK293T and MSC-3H cells showing GFP-MXD1 (green) and EU-labelled RNA (red). Scale bars: 10 μm.

It is established the effect of MYC as a positive regulator of RNA pol I [27, 30–32] and it has also been reported that MXD1 down-regulates rRNA synthesis throughout the repression of UBF expression in fibroblasts [33]. We analyzed in our models whether MXD1 down-regulates rDNA transcription. We transfected the K562 cells with siRNA against *MXD1* and at 72 h post-transfection we counted the cells and prepare protein lysates and total RNA. We confirmed a decrease in MXD1 protein levels in cells transfected with the siRNA, whereas UBF levels did not change (Figure 8A, left panel). We also tested the effect of MXD1 overexpression on UBF levels. Cell were transduced with lentivirus containing the GFP-MXD1 gene and 5 days after the levels of UBF were determined by immunoblot. The results showed no significant changes in UBF levels upon MXD1 overexpression (Figure 8A, right panel). Next, we determined the amounts of total RNA per cell in MXD1 knock-down and in the control cells. Since rRNA accounts for approximately 80% of total RNA in the cell [20], we assumed that if there was a change in total RNA levels it would be mainly due to ribosomal RNA. As shown in Figure 8B, MXD1 knock-down in K562 cells led to a higher amount of total RNA in the cell as compared with controls. This tendency was observed in the four experiments performed, although the difference with the control cells was not statistically significant (*P* = 0.37). Next we determined the levels of pre-rRNA (45S rRNA) which is the primary and unstable product of RNA pol I transcription, in MXD1-silenced K562 cells. pre-RNA was measured by RT-qPCR using 45S rRNA specific primers and the results showed higher levels of 45S rRNA in cells with decreased MXD1 levels (Figure 8C). We also determined the pre-rRNA levels in HeLa cells transfected with sh-MXD vector. The decrease in MXD1 protein was confirmed by immunoblot (Figure 8D, upper panel). The results showed an elevation of 45S pre-rRNA upon MXD1 silencing (Figure 8D, lower panel). Finally, we tested the rate of the novo rRNA synthesis with pulses of 5-ethynyluridine (EU). The increased rRNA synthesis upon MXD1 depletion in K562 was also observed after labelling this EU incorporation in nascent rRNA with an azide-containing Alexa Fluor594 fluorophore (Figure 8E). In a complementary approach, we tested the effect of MXD1 enforced expression on rRNA synthesis. HeLa and HEK293T cells were transfected with a GFP-MXD1 expression vector, and MSC-3H cells were transduced with GFP-MXD1 lentiviral particles. In the three cell lines GFP-MXD1 was overexpressed 48 h after transfection as shown by immunoblot (Figure 9A). De novo RNA synthesis was determined by EU pulse labelling. The EU signals revealed that those cells with MXD1 overexpression showed a reduced RNA synthesis with respect to controls (Figure 9B, 9C). The EU labelling in the cells transfected or transduced with the empty vector was similar than in the non-transfected cells (not shown).

## DISCUSSION

In this work we report that MXD1 localizes within the nucleolus and binds UBF and rDNA chromatin. These novel findings open new biological activities for MXD1. Nucleolar localization of endogenous MXD1 was observed in growing cells from different tissues origins (carcinoma, leukemia, embryonic stem cells) as well as in primary post-mitotic cells as neurons and Sertoli cells. MXD1 accumulates in the nucleolus although it is also present in the nucleoplasm. However, it is of note that MXD1 nucleolar localization was observed for endogenous MXD1 in proliferating cells, where it is expressed at relative low levels, as compared to quiescent cells. Moreover, when MXD1 levels were elevated after transfection, the MXD1 was strongly accumulated in nucleoli. In contrast, overexpressed MNT, another member of the MXD family, did not accumulate in nucleoli, supporting the specific nucleolar localization of MXD1. Previous reports have also described a nucleolar localization for MYC when expressed at supra-physiological levels or upon treatment with proteasome inhibitors [27, 31, 34]. In contrast, in our experiments we have analysed basal MYC levels in proliferating cells. In this setting MYC is present in the nucleoplasm and excluded from the nucleoli in the cell lines under analysis.

Nucleolar localization of MXD1 led us to explore the possibility of an involvement of MXD1 in rRNA synthesis, the major biological role of nucleolus. In fact, by immunofluorescence MXD1 concentrates in FCs and by immunogold electron microscopy MXD1 distributes in FCs and associated DFC, two functionally linked structures -the FC/DFC unit- where rDNA transcription and initial steps of rRNA processing take place [17, 35].

In fact, inhibition of RNA pol I transcription with a low concentration of actinomycin D, which induces peripheral segregation of both the rRNA genes and UBF [36], also redistributes MXD1 at the periphery of the nucleolus. This suggests that MXD1 and UBF remain associated with inactive rRNA genes. In this vein, it has been already reported that MXD1 downregulates rRNA synthesis through MXD1-dependent downregulation of UBF in mouse fibroblasts [33]. We have confirmed the MXD1-dependent downregulation of rRNA synthesis. However, in K562 cells we could not detect a significant change in UBF levels upon MXD1 depletion or overexpression. Therefore, in our model MXD1 must impair rRNA synthesis through an alternative mechanism, although a small effect on UBF may persist. It is conceivable that MXD1 impairs UBF function in rDNA transcription through the MXD1-UBF interaction. Actually, the fact that MXD1 binds not only the promoter but also the IGS in the rDNA chromatin supports an indirect involvement of MXD1 in the rDNA transcription regulation rather than a direct repressive effect upon binding to E-boxes. So it cannot be ruled out the possibility that MXD1 binds to rDNA through another DNA-bound protein. The detailed molecular mechanisms of this interaction require further work to be unveiled.

The ability of MYC to stimulate ribosome biogenesis is critical for the orchestration of the processes of cell cycling and energy production required for proliferation of cancer cells [37]. Our work suggests that MXD1 antagonizes MYC in its positive regulation of rRNA synthesis. rRNA and ribosomal protein synthesis contribute to MYC oncogenic activity. Indeed, the inhibition of MYC activity on RNA pol I has been proposed as a therapeutic target [38]. We hypothesize that a major role of MXD1 could be to curb excessive MYC activity on ribosome biosynthesis and cell growth. Further work is required to demonstrate these hypotheses.

## MATERIALS AND METHODS

### Cell culture transfections and lentiviral transduction

Human K562 chronic myeloid leukemia, HEK293T embryonic kidney and HeLa cervical cancers cells, were from ATCC and grown in RPMI (K562) or DMEM (other cell lines) with fetal calf serum and antibiotics. MSC-3H cells, transformed human mesenchymal stem cells, were grown as described [39]. Human embryonic stem cells HS181 [40] were grown using mTeSR1 medium (StemCell Technologies) on Geltrex-coated plates (ThermoFisher). Transient transfections were carried out using the jetPEI reagent (Polyplus Transfection) or with Ingenio Electroporation solution (Mirus) in an Amaxa nucleofector. For lentivirus production, HEK293T cells were transfected using the jetPEI reagent method with virion packaging vectors (pCMV-VSV-G and psPAX2) and the GFP-MXD1 lentiviral construct or its empty vector in a ratio amount of 1:3:4 respectively. Two days after transfection, supernatant containing lentiviral particles was collected and filtered through 0.45 μm pore size filters (Merck Millipore). Viral particles were concentrated by precipitation using PEG8000 and resuspended in serum-free DMEM. K562 and MSC-3H cells were infected with a MOI of 2 in presence of 5 μg/ml of polybrene for 48 h. The plasmids and siRNAs used are described in Supplementary Table S1

### Immunoblot and immunoprecipitation

Cell lysis, immunoblots and immunoprecipitations were performed as described elsewhere [41]. The primary antibodies used are described in Supplementary Table S2 and the immunoblots were revealed with an Odyssey scanner (Li-Cor Biosciences).

### Immunofluorescence and immunogold staining

Tissue fragments from rat testes and trigeminal ganglion were fixed with 4% paraformaldehyde in PBS. Squash preparations from both tissues were processed and immunostained as reported previously [23]. The protocol was approved by the Ethics Committee of the University of Cantabria. Adherent cells grown on glass coverslips and were fixed with 4% paraformaldehyde in PBS for 15 min at room temperature. Fixed cells were washed with PBS and permeabilized with 1% Triton X-100 in PBS during 30 min. Then, cells were treated with blocking buffer (3% BSA; 0.1% Triton X-100 in PBS) for 20 min, washed with PBS and 0.1% Triton X-100 in PBS, and incubated overnight at 4°C with the primary antibodies diluted in blocking buffer. The slides were incubated for 1 h at room temperature with the secondary antibody conjugated with FITC or Cy3 (Jackson Laboratories). The samples were mounted with ProLong Gold Antifade mountant (LifeTechnologies). Confocal images were obtained with a Leica TCS SP5 microscope and processed using Adobe Photoshop and ImageJ (http://rsb.info.nih.gov/ij/) softwares. For immunogold electron microscopy, tissue fragments from sensory ganglion were fixed with 4% paraformaldehyde and embedded in Lowicryl 4 M. Ultrathin sections were immunolabeled with the anti-MXD1 antibody and then with a secondary antibody conjugated with 10 nm gold particles The antibodies used are described in the Supplementary Table S2.

### *In situ* proximity ligation assay

*In situ* Proximity Ligation Assay (PLA) was performed with Duolink *in situ* Red Starter kit Mouse/Rabbit (Sigma-Aldrich) according to manufacturer's instructions with home-made buffers as previously described [41]. *In situ* PLA positive signals were quantified using the ImageJ software. At least 200 nuclei were measured for each experimental condition. Cell samples were visualized using a confocal microscope as described above. The primary antibodies used are described in Supplementary Table S2.

### RNA analysis

Total RNA was isolated using the RNeasy kit (Qiagen). cDNA was generated by reverse transcription (RT) using the iScript (Bio-Rad). Quantitative polymerase chain reaction (qPCR) was performed with specific primers (Supplementary Table S3) using the iQSYBR Green supermix (Bio-Rad). Labelling of rRNA with 5-ethynyluridine (EU) was performed with the Click-iT RNA AlexaFluor594 kit (ThermoFisher) for 15 min (K562 cells) or 50 min (HeLa, HEK293T and MSC-3H cells) and assessed by microscopic imaging. Levels of mRNA and pre-rRNA were normalized against actin and RPS14 mRNA levels.

### Chromatin immunoprecipitation

Chromatin immunoprecipitation (ChIP) was performed essentially as described [41]. Immunoprecipitated DNA was purified with the QIAquick PCR Purification Kit (Qiagen) and analysed by qPCR. The antibodies and primers used are described in Table 2 and Table 3 respectively.

## SUPPLEMENTARY MATERIALS


