# Supplementary material for: MXD1 localizes in the nucleolus, binds UBF and impairs rRNA synthesis
Source: Oncotarget. 2016 Aug 31;7(43):69536–48. doi: 10.18632/oncotarget.11766 (PMC5342496; doi:10.18632/oncotarget.11766)
Supplement: Supplementary file 1 [file oncotarget-07-69536-s001.pdf]

MXD1 localizes in the nucleolus, binds UBF and impairs rRNA synthesis

Supplementary Materials

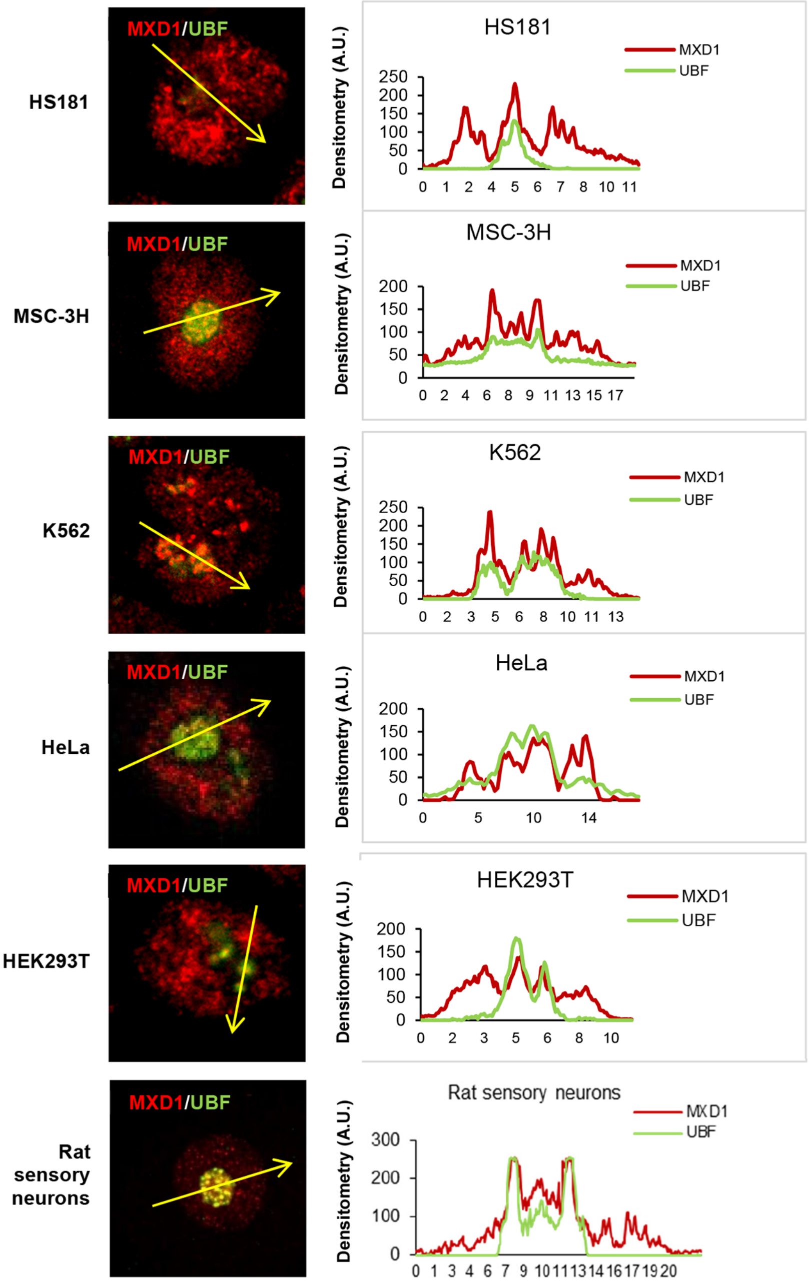

Supplementary Figure S1: Co-localization of MXD1 and UBF in fibrillar centers. Fluorescence intensities line profiles of representative cells shown in Figure 1B for MXD1 (red) and UBF (green) across the nucleolus revealed their co-localization in the fibrillar centers of the nucleolus. Images were acquired with a confocal microscope and analyzed with the ImageJ software.

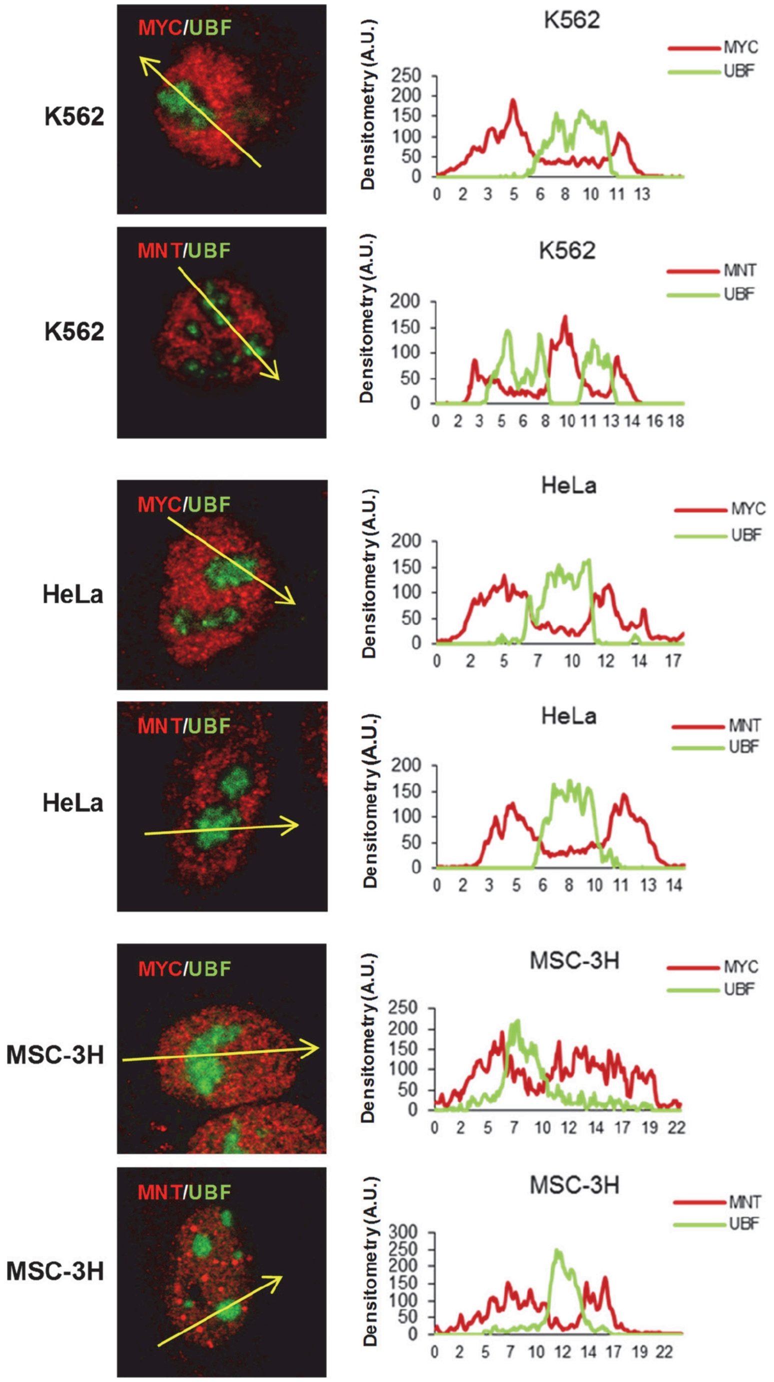

Supplementary Figure S2: Lack of co-localization of UBF with MYC or MNT in human cell lines. Fluorescence intensities of MYC or MNT (red) and UBF (green) line profiles of representative cells in Figure 5 illustrating the lack of co-localization of MYC or MNT with UBF within the nucleolus. Images were acquired with a confocal microscope and analyzed with the ImageJ software.

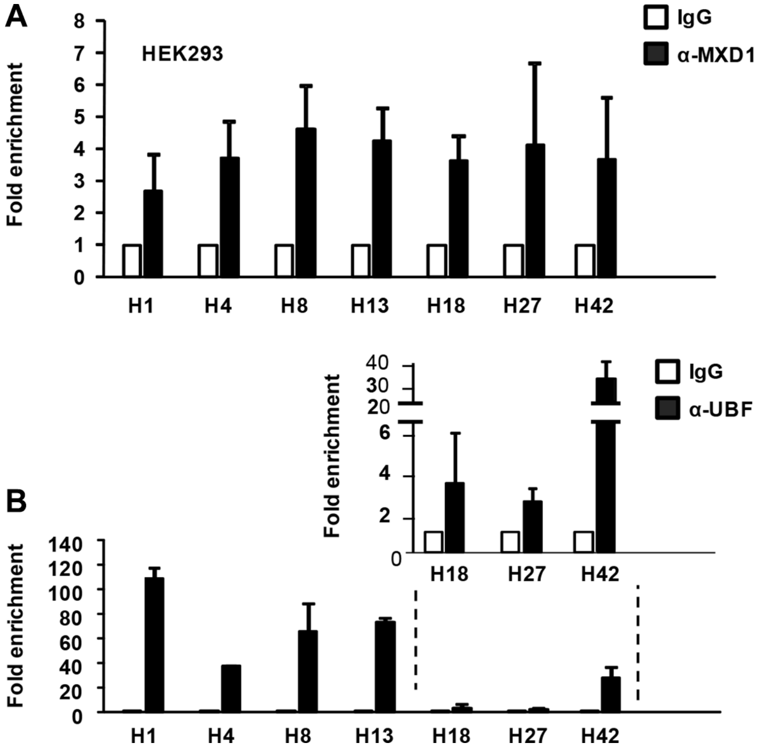

Supplementary Figure S3: MXD1 binding to rDNA chromatin in HEK293T cells. ChIP of MXD1 and UBF in HEK293T cells. The amplicons H1-H42 cover different regions of the rDNA gene and intergenic regions [27]. Data are mean values from four ChIP experiments. The inset shows the signals of UBF in the H18-H42 amplicons.

Supplementary Table S1: Plasmids and siRNAs used in this study

Name	Gene	Origin (Reference)
pCMV-VSV-G	VSV-G gene encoding enveloped lentiviral protein	Didier Trono (Addgene 8454)
psPAX2	GAG and POL genes enconding packaging lentiviral proteins	Robert A. Weinberg (Addgene 12260)
pReceiver-Lv103-GFP-MXD1	human GFP-MXD1 fusion gene	Genecopoeia (EX-F0141-Lv103)
pReceiver-Lv103-GFP-MNT	human GFP-MNT fusion gene	Genecopoeia (EX-F0141-Lv103)
pReceiver-Lv103	empty vector expressing the GFP gene	Genecopoeia (EX-Mm20333-Lv103)
pLKO.1-sh-MXD1	short hairpin RNA TRCN0000017571 against human MXD1 mRNA	Sigma- MISSION (SHCLNG-NM_002357)
pLKO.1 control	Empty vector	Sigma- MISSION (SHC001)
siRNA Universal Negative Controls	scramble RNA sequence with no homology to known gene sequences	Sigma- MISSION (SIC001)
MXD1 siRNA (1)	siRNA against human MXD1 mRNA	Sigma- MISSION (SASI_Hs01_00179640)
MXD1 siRNA (2)	siRNA against human MXD1 mRNA	Sigma- MISSION (SASI_Hs01_00179641)

Supplementary Table S2: Antibodies used in this study

Antibody	Immunogen (species)	Type	Source	Technique and dilution
Anti-β-actin	C-terminus (human)	Goat polyclonal	Santa Cruz Biotech (I-19, sc-1616)	IB (1:1000);
anti- ɣ Hemoglobin	Full length (human)	Mouse monoclonal	Santa Cruz Biotech. (51-7, sc-21756)	isPLA (1:200)
Anti-β Hemoglobin	Amino acids 67-147 (human)	Rabbit polyclonal	Santa Cruz Biotech. (sc-21006)	isPLA (1:200)
Anti-MNT	Amino acids 226-361 (human)	Rabbit polyclonal	Santa Cruz Biotech. (M-132, sc-769)	IB (1:1000); IF (1:50)
Anti-MXD1	C-terminal (human)	Rabbit polyclonal	Santa Cruz Biotech. (C-19, sc-222)	WB (1:1000); IF (1:50); isPLA (1:200) IP; ChIP
Anti-MYC	Amino acids 1-262 (human)	Rabbit polyclonal	Santa Cruz Biotech. (N-262, sc-21756)	IB (1:1000); IF (1:50); isPLA (1:200)
Anti-α-tubulin	Amino acids149-448 (human)	Rabbit polyclonal	Santa Cruz Biotech. (H300, sc-5546)	IB (1:1000);
Anti-UBF	Amino acids 1-220 (human)	Mouse monoclonal	Santa Cruz Biotech. (F-9, sc-13125)	IF (1:50); isPLA (1:200)
Anti-UBF	Amino acids 1-220 aa (human)	Rabbit polyclonal	Santa Cruz Biotech. (H-300, sc-9131)	IB (1:1000); IP; ChIP
Secondary antibodies				
Anti-Rabbit Cy3-conjugated	Rabbit heavy and light IgG	Goat polyclonal	Jackson InmunoResearch laboratories Inc. (111-165-144)	IF (1:800)
Anti-Mouse Texas Red- conjugated	Mouse heavy and light IgG	Goat polyclonal	Jackson InmunoResearch laboratories Inc. (111-075-045)	IF (1:100)
Anti-Mouse FITC-conjugated	Mouse heavy and light IgG	Goat polyclonal	Jackson InmunoResearch laboratories Inc. (111-096-003)	IF (1:100)
Anti-rabbit coupled to 10 nm gold particles	Rabbit heavy and light IgG	Goat polyclonal	BioCell UK	Immuno electron microscopy (1:50)

IB, immunoblot. IF, immunofluorescence; ChIP, chromatin immunoprecipitation; isPLA, *in situ* Proximity Ligase Assay.

Supplementary Table S3: Primers used in this study and the amplicon names

Gene	Amplicon	Primers sequence (5′→3′)	Tm (°C)	Use
Chromosome 13 intergenic region	chr13	GAGGAAGCCTGCACACCTAC<br>AATTCAACAGCGAGGGGTAA	57	ChIP
Chromosome 15 intergenic region	chr15	TGCTGAGGGTTCAAACTGTG<br>CCTCCAGAACCACAGCAGAT	57	ChIP
rDNA, transcribed region 45S	H1	GGCGGTTTGAGTGAGACGAGA<br>ACGTGCGCTCACCGAGAGCAG	63	ChIP
rDNA, transcribed region, 18S	H4	CGACGACCCATTCGAACGTCT<br>CTCTCCGGAATCGAACCCTGA	63	ChIP
rDNA transcribed region, 5.8S	H8	AGTCGGGTTGCTTGGGAATGC<br>CCCTTACGGTACTTGTTGACT	63	ChIP
rDNA, non-transcribed region	H13	ACCTGGCGCTAAACCATTCGT<br>GGACAAACCCTTGTGTCGAGG	63	ChIP
rDNA, non-transcribed region	H18	GTTGACGTACAGGGTGGACTG<br>GGAAGTTGTCTTCACGCCTGA	63	ChIP
rDNA, non-transcribed region	H27	CCTTCCACGAGAGTGAGAAGCG<br>CTCGACCTCCCGAAATCGTACA	63	ChIP
rDNA, non-transcribed region	H32	GGAGTGCGATGGTGTGATCT<br>TAAAGATTAGCTGGGCGTGG	63	ChIP
rDNA, non-transcribed region	H42	AGAGGGGCTGCGTTTTCGGCC<br>CGAGACAGATCCGGCTGGCAG	63	ChIP
rDNA, 5′ transcribed region	45S	GTCCCCTCGTCTCTCCTCTC<br>CAAGTCGACAACCACTGGAG	57	RT-qPCR
MXD1	MXD1	ACCCGAATCAAGTCGACACAC<br>ATCCTCTCAATGCCCAGCTTC	57	RT-qPCR
ACTB<br>(β-Actin)	Actin	AAAATCTGGCACCACACCTTC<br>TAGCACAGCCTGGATAGCAA	57	RT-qPCR
TBP	TBP	CCACAGCTCTTCCACTCACA<br>GGATTATATTCGGCGTTTCG	57	RT-qPCR
RPS14	S14	TCACCGCCCTACACATCAAACT<br>CTGCGAGTGCTGTCAGAGG	57	RT-qPCR

All genes are human. ChIP, chromatin immunoprecipitation; RT-qPCR, reverse transcription-quantitative polymerase chain reaction. Tm, annealing temperature in the PCR reaction. The H4-H42 amplicons are as described [27]. The forward primer is shown in the upper line.
